# Supplementary figures and images for: Computational insights into flavonoids inhibition of dengue virus envelope protein: ADMET profiling, molecular docking, dynamics, PCA, and end-state free energy calculations
Source: PLoS One. 2025 Jul 9;20(7):e0327862. doi: 10.1371/journal.pone.0327862 (PMC12240381; doi:10.1371/journal.pone.0327862)

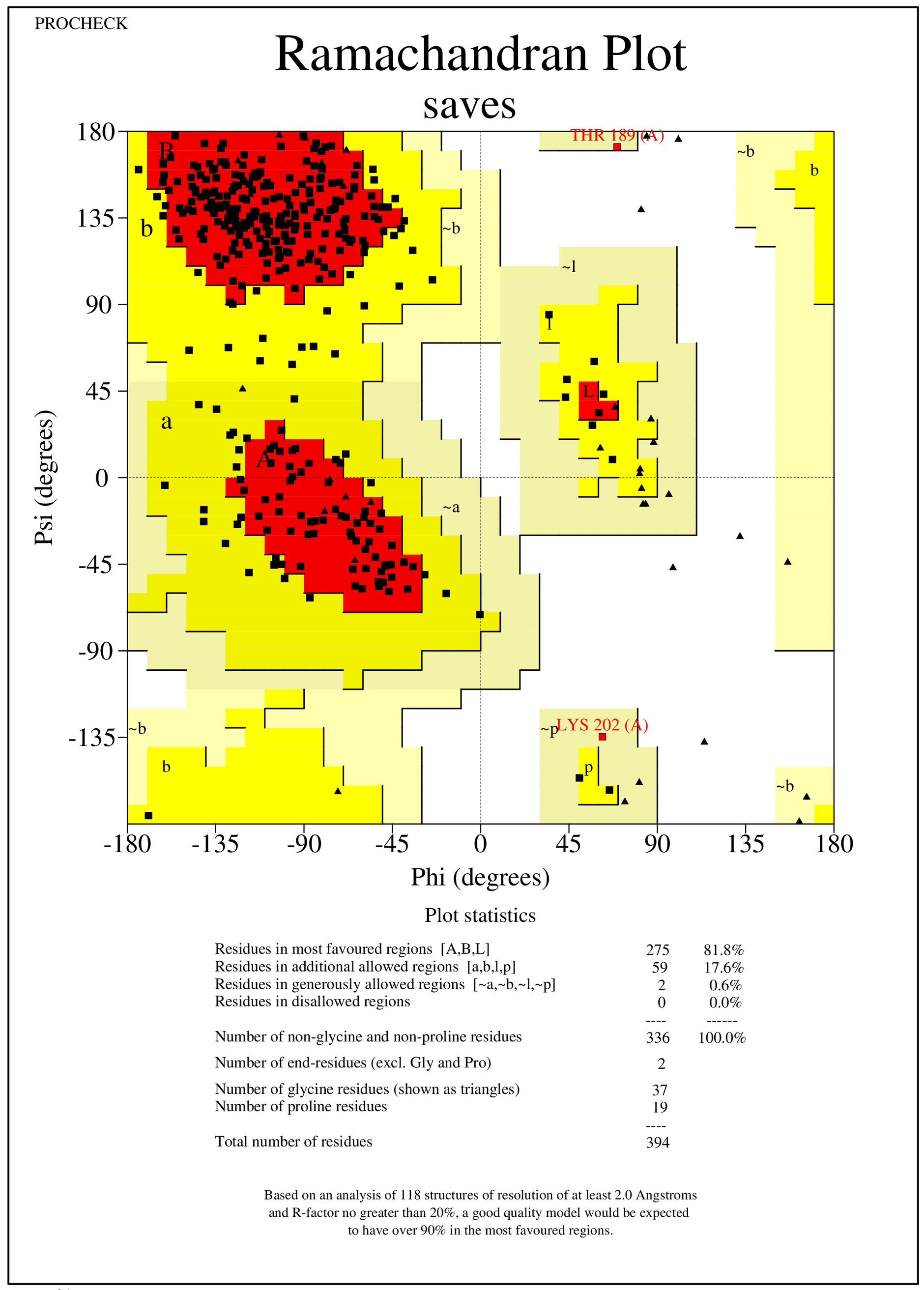

Supplement: S1 Fig — (TIF) [file pone.0327862.s001.tif]

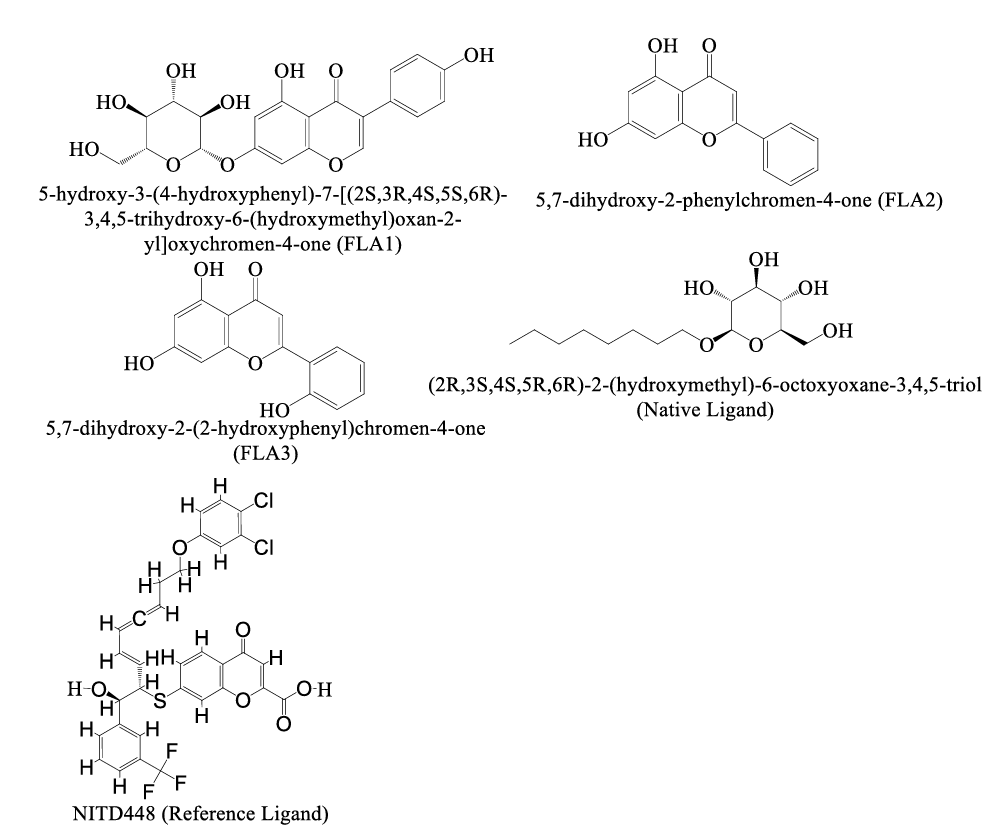

Supplement: S2 Fig — (TIF) [file pone.0327862.s002.tif]

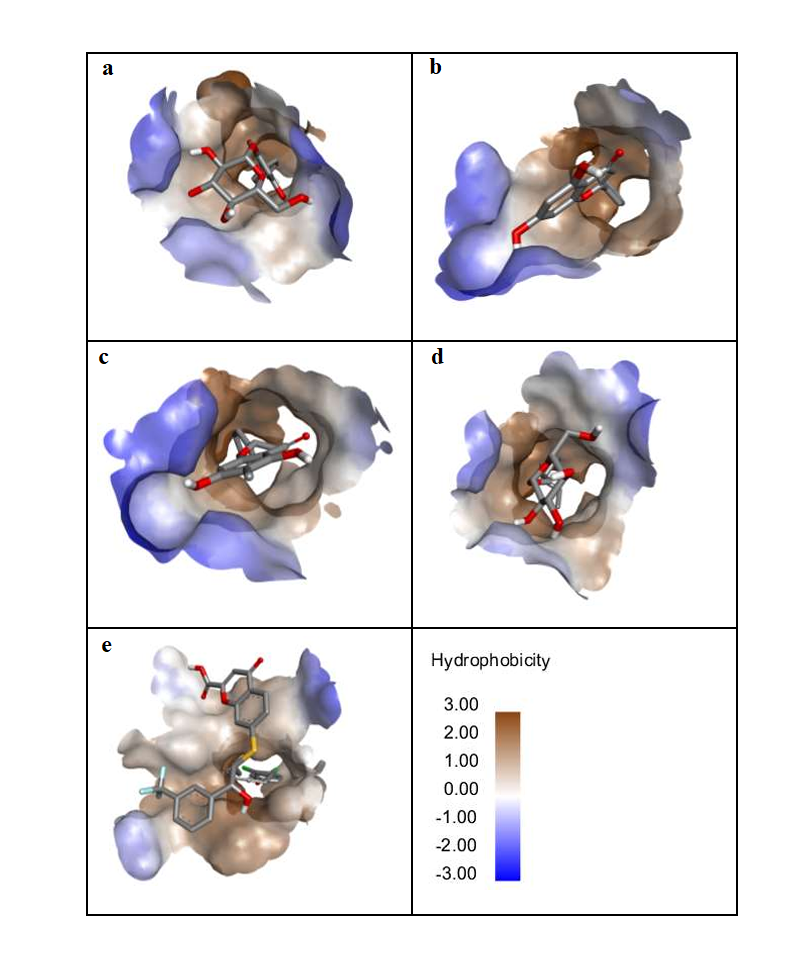

Supplement: S3 Fig — (TIF) [file pone.0327862.s003.tif]

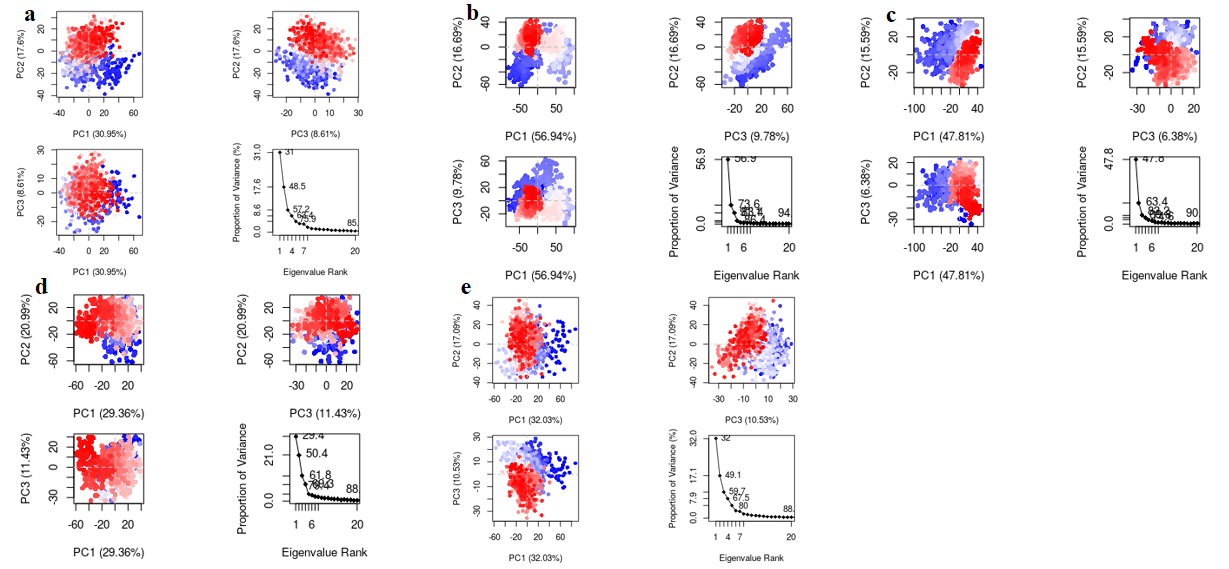

Supplement: S4 Fig — (TIF) [file pone.0327862.s004.tif]

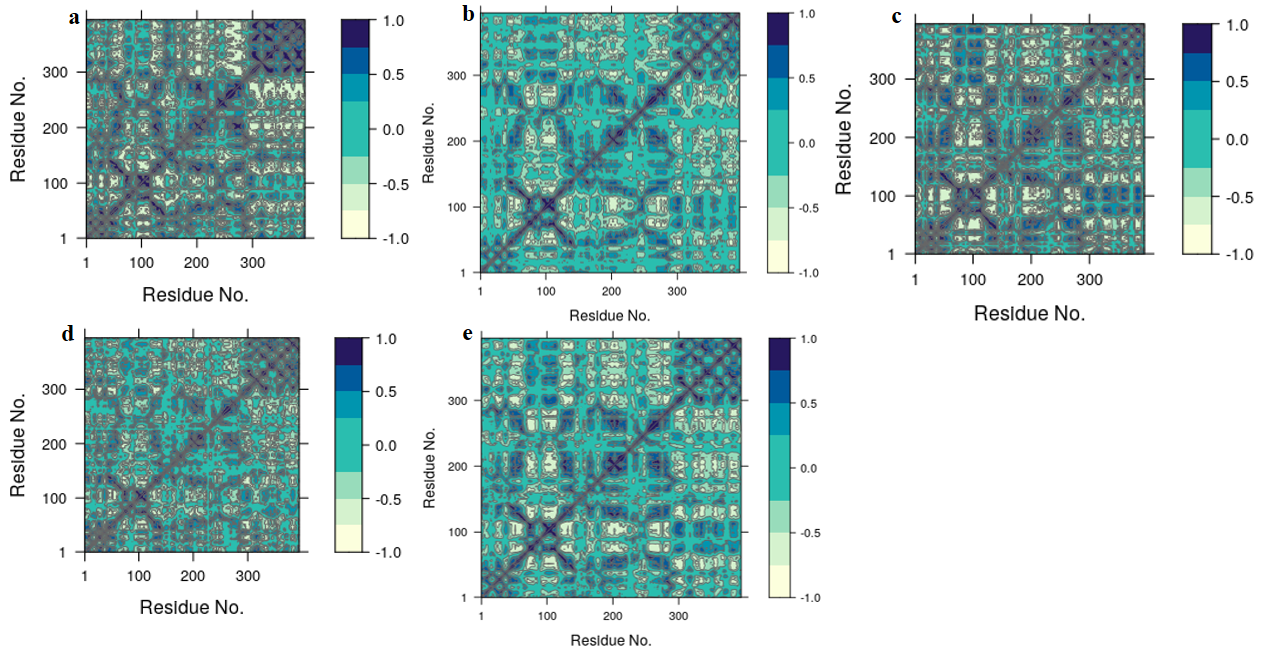

Supplement: S5 Fig — (TIF) [file pone.0327862.s005.tif]

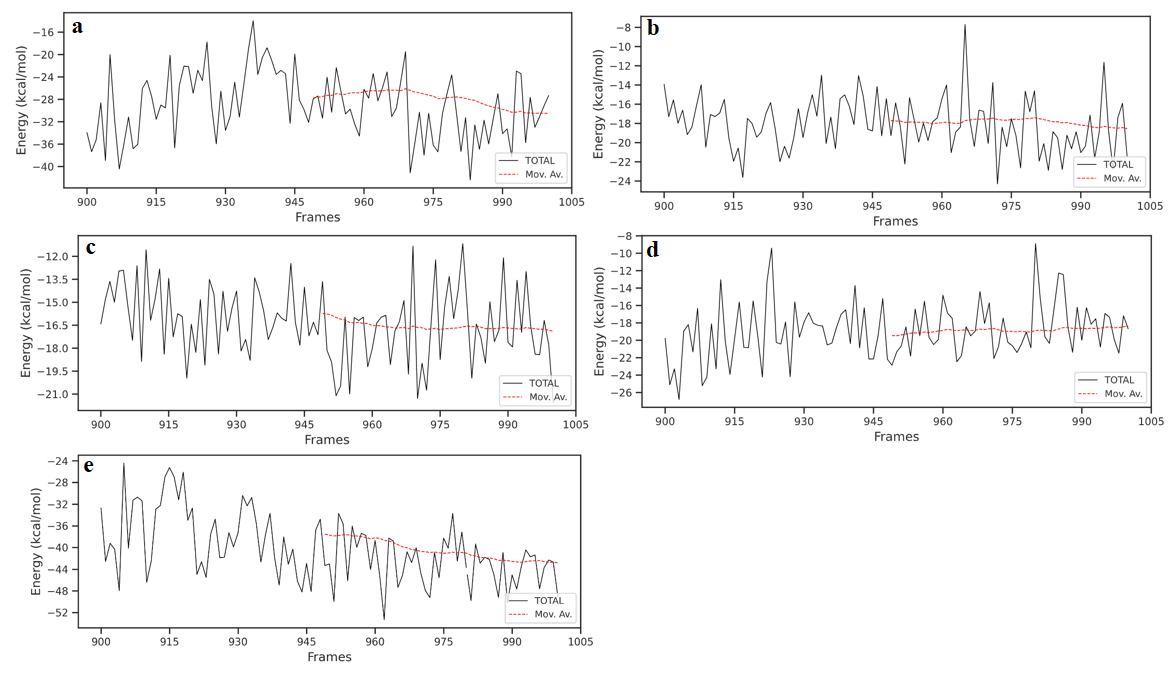

Supplement: S6 Fig — (TIF) [file pone.0327862.s006.tif]

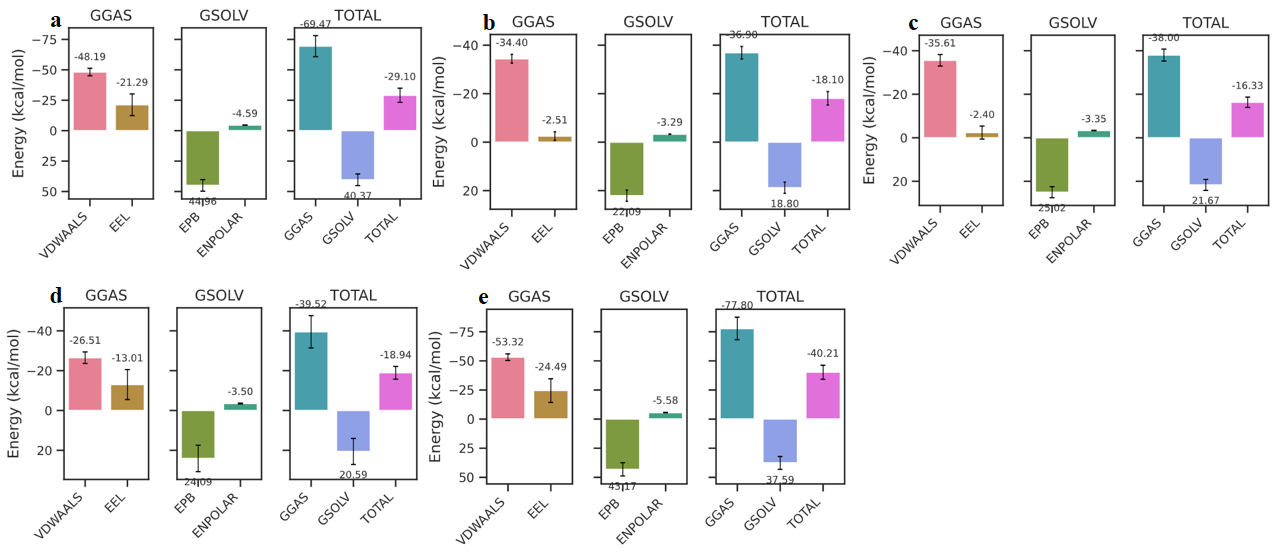

Supplement: S7 Fig — (a) FLA1, (b) FLA2, (c) FLA3, (d) native ligand, (e) Reference ligand, from the last 20 ns stable trajectories of the protein-ligand complexes. (TIF) [file pone.0327862.s007.tif]

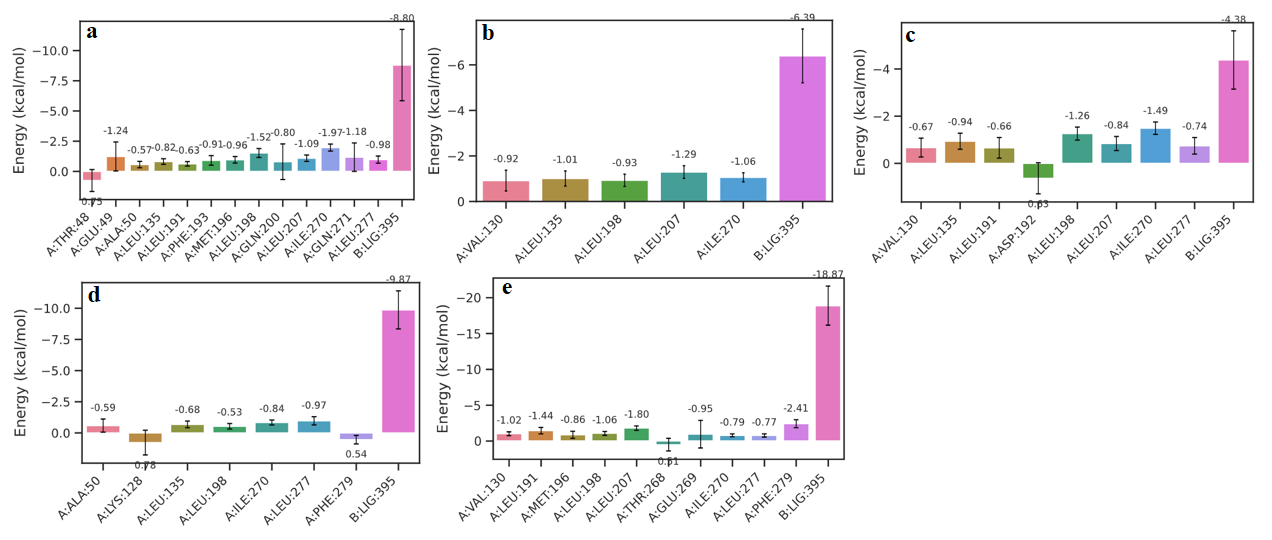

Supplement: S8 Fig — (TIF) [file pone.0327862.s008.tif]

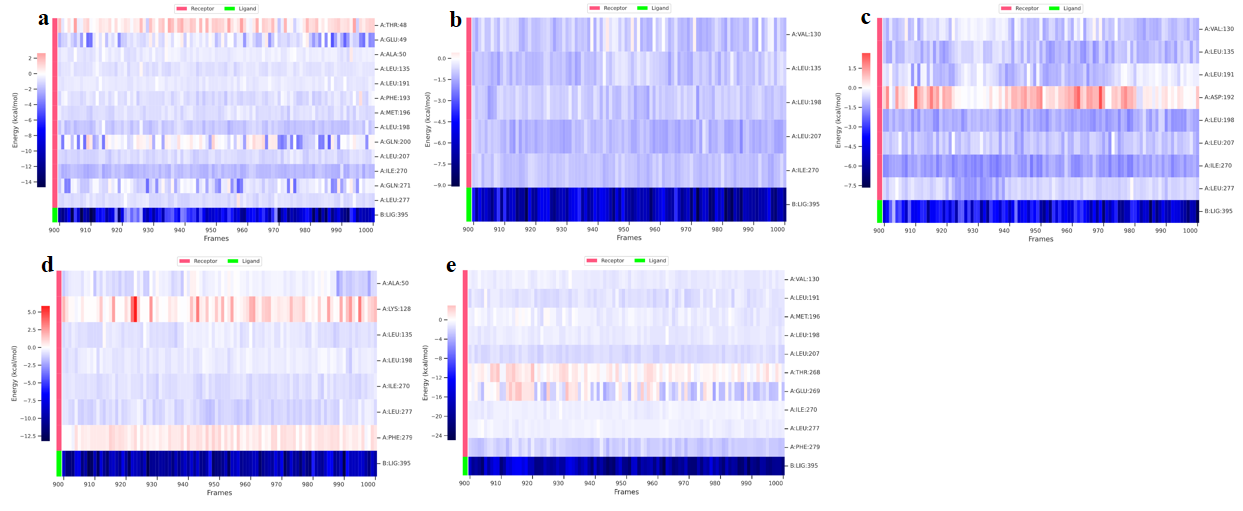

Supplement: S9 Fig — A heat-map showing residue-wise contributions per frame of the simulation. (TIF) [file pone.0327862.s009.tif]
