# Supplementary material for: Computational insights into flavonoids inhibition of dengue virus envelope protein: ADMET profiling, molecular docking, dynamics, PCA, and end-state free energy calculations
Source: PLoS One. 2025 Jul 9;20(7):e0327862. doi: 10.1371/journal.pone.0327862 (PMC12240381; doi:10.1371/journal.pone.0327862)
Supplement: S1 Table — (DOCX) [file pone.0327862.s010.docx]

**S1 Table:** Toxicity from Protox-III

| **Compounds** | **Hepatotoxicity** | **Carcinogenicity** | **Immunotoxicity** | **Mutagenicity** | **Cytotoxicity** |
| --- | --- | --- | --- | --- | --- |
| FLA1 | Inactive | Inactive | Inactive | Inactive | Inactive |
| FLA2 | Inactive | Inactive | Inactive | Inactive | Inactive |
| FLA3 | Inactive | Inactive | Inactive | Inactive | Inactive |
| FLA4 | Inactive | Inactive | Inactive | Inactive | Inactive |
| FLA5 | Inactive | Inactive | Inactive | Inactive | Inactive |
| FLA6 | Inactive | Inactive | Inactive | Inactive | Inactive |
| FLA7 | Inactive | Inactive | Inactive | Inactive | Inactive |
| FLA8 | Inactive | Inactive | Inactive | Inactive | Inactive |
| FLA9 | Inactive | Inactive | Inactive | Inactive | Inactive |
| FLA10 | Inactive | Inactive | Inactive | Inactive | Inactive |
| FLA11 | Inactive | Inactive | Inactive | Inactive | Inactive |
| FLA12 | Inactive | Inactive | Inactive | Inactive | Inactive |
| FLA13 | Inactive | Inactive | Inactive | Inactive | Inactive |
| FLA14 | Inactive | Inactive | Inactive | Inactive | Inactive |
| FLA15 | Inactive | Inactive | Inactive | Inactive | Inactive |
| FLA16 | Inactive | Inactive | Inactive | Inactive | Inactive |
| FLA17 | Inactive | Inactive | Inactive | Inactive | Inactive |
| FLA18 | Inactive | Inactive | Inactive | Inactive | Inactive |
| FLA19 | Inactive | Inactive | Inactive | Inactive | Inactive |
| FLA20 | Inactive | Inactive | Inactive | Inactive | Inactive |
| FLA21 | Inactive | Inactive | Inactive | Inactive | Inactive |
| FLA22 | Inactive | Inactive | Inactive | Inactive | Inactive |
| FLA23 | Inactive | Inactive | Inactive | Inactive | Inactive |
| FLA24 | Inactive | Inactive | Inactive | Inactive | Inactive |
| FLA25 | Inactive | Inactive | Inactive | Inactive | Inactive |
| FLA26 | Inactive | Inactive | Inactive | Inactive | Inactive |
| FLA27 | Inactive | Inactive | Inactive | Inactive | Inactive |
| FLA28 | Inactive | Inactive | Inactive | Inactive | Inactive |
| FLA29 | Inactive | Inactive | Inactive | Inactive | Inactive |
| FLA30 | Inactive | Inactive | Inactive | Inactive | Inactive |
| FLA31 | Inactive | Inactive | Inactive | Inactive | Inactive |
| FLA32 | Inactive | Inactive | Inactive | Inactive | Inactive |
| FLA33 | Inactive | Inactive | Inactive | Inactive | Inactive |
| Native ligand | Inactive | Inactive | Inactive | Inactive | Inactive |
| Reference ligand | Inactive | Inactive | active | Inactive | Inactive |
