# Supplementary material for: Computational insights into flavonoids inhibition of dengue virus envelope protein: ADMET profiling, molecular docking, dynamics, PCA, and end-state free energy calculations
Source: PLoS One. 2025 Jul 9;20(7):e0327862. doi: 10.1371/journal.pone.0327862 (PMC12240381; doi:10.1371/journal.pone.0327862)
Supplement: S2 Table — (DOCX) [file pone.0327862.s011.docx]

**S2 Table: Drug-Likeness Properties of hit candidates and reference drug through Swiss ADME Server**

| **PubChem CID** | **Molecular weight (g/mol)** | **No. of rotatable bonds** | **No. of H bond acceptors** | **No. of H bond donors** | **TPSA (Å^2^)** | **MLOGP** | **molar refractivity** | **Lipinski’s rule of five** | **Ghose filter** | **Veber rule** |
| --- | --- | --- | --- | --- | --- | --- | --- | --- | --- | --- |
| **FLA1** | 432.38 | 4 | 10 | 6 | 170.05 | -1.61 | 106.11 | Yes; 1 violation | Yes | No; 1 violation |
| **FLA2** | 254.24 | 1 | 4 | 2 | 70.67 | 1.08 | 71.97 | Yes; 0 violation | Yes | Yes |
| **FLA3** | 270.28 | 1 | 4 | 2 | 66.76 | 1.52 | 74.51 | Yes; 0 violation | Yes | Yes |
| **Reference ligand** | 663.49 | 12 | 9 | 2 | 122.27 | 4.84 | 164.06 | No;2 violations | No; 3 violations | No; 1 violation |
