# Supplementary material for: Computational insights into flavonoids inhibition of dengue virus envelope protein: ADMET profiling, molecular docking, dynamics, PCA, and end-state free energy calculations
Source: PLoS One. 2025 Jul 9;20(7):e0327862. doi: 10.1371/journal.pone.0327862 (PMC12240381; doi:10.1371/journal.pone.0327862)
Supplement: S3 Table — (DOCX) [file pone.0327862.s012.docx]

**S3 Table:** ADMET properties from ADMETlab 2.0

| **Compounds** | **LogS** | **HIA** | **BBB** | **PPB** | **CYP1A2-inh** | **CYP1A2-sub** | **CYP2C19-inh** | **CYP2C19-sub** |
| --- | --- | --- | --- | --- | --- | --- | --- | --- |
| FLA1 | -3.896 | 0.659 | 0.092 | 90.25% | 0.065 | 0.041 | 0.022 | 0.057 |
| FLA2 | -3.506 | 0.012 | 0.02 | 98.03% | 0.989 | 0.262 | 0.736 | 0.06 |
| FLA3 | -3.489 | 0.019 | 0.012 | 98.15% | 0.987 | 0.21 | 0.496 | 0.053 |
| FLA4 | -3.649 | 0.006 | 0.138 | 97.29% | 0.835 | 0.696 | 0.863 | 0.214 |
| FLA5 | -3.491 | 0.007 | 0.02 | 98.59% | 0.976 | 0.126 | 0.3 | 0.053 |
| FLA6 | -3.608 | 0.01 | 0.017 | 97.06% | 0.975 | 0.853 | 0.634 | 0.069 |
| FLA7 | -3.635 | 0.007 | 0.016 | 97.86% | 0.966 | 0.109 | 0.543 | 0.051 |
| FLA8 | -3.606 | 0.015 | 0.012 | 97.25% | 0.988 | 0.145 | 0.588 | 0.051 |
| FLA9 | -3.624 | 0.008 | 0.009 | 97.86% | 0.972 | 0.11 | 0.181 | 0.046 |
| FLA10 | -3.709 | 0.032 | 0.007 | 95.93% | 0.98 | 0.867 | 0.408 | 0.056 |
| FLA11 | -3.704 | 0.032 | 0.007 | 96.04% | 0.982 | 0.879 | 0.463 | 0.055 |
| FLA12 | -3.631 | 0.017 | 0.004 | 96.96% | 0.971 | 0.824 | 0.391 | 0.059 |
| FLA13 | -2.99 | 0.096 | 0.025 | 92.06% | 0.219 | 0.295 | 0.037 | 0.056 |
| FLA14 | -4.04 | 0.74 | 0.032 | 88.90% | 0.06 | 0.03 | 0.033 | 0.046 |
| FLA15 | -4.04 | 0.74 | 0.032 | 88.90% | 0.06 | 0.03 | 0.033 | 0.046 |
| FLA16 | -3.812 | 0.864 | 0.061 | 87.42% | 0.087 | 0.048 | 0.017 | 0.054 |
| FLA17 | -3.478 | 0.005 | 0.019 | 97.60% | 0.978 | 0.811 | 0.888 | 0.058 |
| FLA18 | -2.725 | 0.012 | 0.029 | 82.66% | 0.056 | 0.096 | 0.065 | 0.075 |
| FLA19 | -2.698 | 0.011 | 0.093 | 97.39% | 0.99 | 0.194 | 0.482 | 0.068 |
| FLA20 | -3.464 | 0.008 | 0.054 | 97.08% | 0.976 | 0.127 | 0.808 | 0.054 |
| FLA21 | -2.99 | 0.096 | 0.025 | 92.06% | 0.219 | 0.295 | 0.037 | 0.056 |
| FLA22 | -3.742 | 0.005 | 0.153 | 96.87% | 0.873 | 0.665 | 0.851 | 0.159 |
| FLA23 | -3.44 | 0.01 | 0.02 | 97.56% | 0.981 | 0.132 | 0.59 | 0.048 |
| FLA24 | -3.663 | 0.009 | 0.009 | 97.71% | 0.971 | 0.677 | 0.511 | 0.055 |
| FLA25 | -3.497 | 0.005 | 0.02 | 97.97% | 0.978 | 0.8 | 0.836 | 0.059 |
| FLA26 | -3.345 | 0.48 | 0.06 | 95.54% | 0.964 | 0.194 | 0.791 | 0.058 |
| FLA27 | -3.878 | 0.627 | 0.074 | 87.82% | 0.07 | 0.037 | 0.016 | 0.053 |
| FLA28 | -3.786 | 0.158 | 0.004 | 91.48% | 0.954 | 0.953 | 0.187 | 0.063 |
| FLA29 | -4.421 | 0.017 | 0.034 | 87.27% | 0.957 | 0.966 | 0.794 | 0.295 |
| FLA30 | -3.847 | 0.019 | 0.005 | 96.12% | 0.975 | 0.786 | 0.344 | 0.057 |
| FLA31 | -4.256 | 0.007 | 0.021 | 89.39% | 0.953 | 0.954 | 0.886 | 0.201 |
| FLA32 | -3.781 | 0.012 | 0.012 | 96.63% | 0.975 | 0.812 | 0.588 | 0.066 |
| FLA33 | -4.962 | 0.005 | 0.079 | 77.28% | 0.864 | 0.971 | 0.857 | 0.689 |
| Native ligand | -1.554 | 0.886 | 0.282 | 71.32% | 0.013 | 0.121 | 0.012 | 0.172 |
| Reference ligand | -4.973 | 0.004 | 0.088 | 99.18% | 0.001 | 9.30E | 0.005 | 5.03E |
