# Supplementary material for: Computational insights into flavonoids inhibition of dengue virus envelope protein: ADMET profiling, molecular docking, dynamics, PCA, and end-state free energy calculations
Source: PLoS One. 2025 Jul 9;20(7):e0327862. doi: 10.1371/journal.pone.0327862 (PMC12240381; doi:10.1371/journal.pone.0327862)
Supplement: S4 Table — (DOCX) [file pone.0327862.s013.docx]

**S4 Table:** Toxicity from ADMETlab 2.0

| **Compounds** | **hERG** | **ROA** | **SkinSen** | **EC** | **EI** | **Respiratory** |
| --- | --- | --- | --- | --- | --- | --- |
| FLA1 | 0.034 | 0.082 | 0.109 | 0.003 | 0.02 | 0.029 |
| FLA2 | 0.037 | 0.088 | 0.926 | 0.016 | 0.948 | 0.513 |
| FLA3 | 0.084 | 0.055 | 0.913 | 0.019 | 0.948 | 0.241 |
| FLA4 | 0.024 | 0.818 | 0.867 | 0.004 | 0.931 | 0.663 |
| FLA5 | 0.043 | 0.242 | 0.837 | 0.013 | 0.927 | 0.179 |
| FLA6 | 0.067 | 0.125 | 0.899 | 0.005 | 0.918 | 0.447 |
| FLA7 | 0.03 | 0.344 | 0.792 | 0.005 | 0.921 | 0.089 |
| FLA8 | 0.057 | 0.057 | 0.928 | 0.011 | 0.945 | 0.266 |
| FLA9 | 0.07 | 0.156 | 0.856 | 0.009 | 0.929 | 0.09 |
| FLA10 | 0.04 | 0.036 | 0.917 | 0.008 | 0.932 | 0.368 |
| FLA11 | 0.044 | 0.035 | 0.919 | 0.006 | 0.929 | 0.433 |
| FLA12 | 0.099 | 0.073 | 0.906 | 0.004 | 0.924 | 0.241 |
| FLA13 | 0.022 | 0.467 | 0.945 | 0.003 | 0.903 | 0.107 |
| FLA14 | 0.028 | 0.051 | 0.034 | 0.003 | 0.01 | 0.06 |
| FLA15 | 0.028 | 0.051 | 0.034 | 0.003 | 0.01 | 0.06 |
| FLA16 | 0.027 | 0.034 | 0.346 | 0.003 | 0.031 | 0.061 |
| FLA17 | 0.058 | 0.212 | 0.86 | 0.022 | 0.95 | 0.231 |
| FLA18 | 0.068 | 0.341 | 0.896 | 0.003 | 0.876 | 0.367 |
| FLA19 | 0.102 | 0.134 | 0.958 | 0.06 | 0.976 | 0.728 |
| FLA20 | 0.072 | 0.333 | 0.895 | 0.019 | 0.97 | 0.101 |
| FLA21 | 0.022 | 0.467 | 0.945 | 0.003 | 0.903 | 0.107 |
| FLA22 | 0.039 | 0.808 | 0.91 | 0.004 | 0.94 | 0.622 |
| FLA23 | 0.048 | 0.322 | 0.926 | 0.05 | 0.962 | 0.087 |
| FLA24 | 0.051 | 0.148 | 0.649 | 0.007 | 0.919 | 0.125 |
| FLA25 | 0.048 | 0.217 | 0.848 | 0.03 | 0.96 | 0.128 |
| FLA26 | 0.053 | 0.457 | 0.942 | 0.017 | 0.969 | 0.129 |
| FLA27 | 0.024 | 0.111 | 0.057 | 0.003 | 0.015 | 0.024 |
| FLA28 | 0.117 | 0.034 | 0.92 | 0.007 | 0.895 | 0.365 |
| FLA29 | 0.052 | 0.063 | 0.649 | 0.004 | 0.471 | 0.58 |
| FLA30 | 0.027 | 0.11 | 0.861 | 0.004 | 0.904 | 0.284 |
| FLA31 | 0.038 | 0.093 | 0.209 | 0.004 | 0.688 | 0.246 |
| FLA32 | 0.02 | 0.122 | 0.858 | 0.005 | 0.882 | 0.481 |
| FLA33 | 0.07 | 0.083 | 0.139 | 0.004 | 0.357 | 0.127 |
| Native ligand | 0.061 | 0.096 | 0.119 | 0.003 | 0.026 | 0.053 |
| Reference ligand | 0.490 | 0.6905 | 0.053 | 7.07E | 0.029 | 0.954 |
