# Supplementary material for: Computational insights into flavonoids inhibition of dengue virus envelope protein: ADMET profiling, molecular docking, dynamics, PCA, and end-state free energy calculations
Source: PLoS One. 2025 Jul 9;20(7):e0327862. doi: 10.1371/journal.pone.0327862 (PMC12240381; doi:10.1371/journal.pone.0327862)
Supplement: S5 Table — (DOCX) [file pone.0327862.s014.docx]

**S5 Table:** Toxicity From pkCSM server

| **Compounds** | **P-glycoprotein substrate** | **P-glycoprotein I Inhibitor** | **P-glycoprotein II inhibitor** | **Renal OCT2 substrate** |
| --- | --- | --- | --- | --- |
| FLA1 | Yes | No | No | No |
| FLA2 | Yes | No | No | No |
| FLA3 | Yes | No | No | No |
| FLA4 | Yes | No | No | No |
| FLA5 | Yes | No | No | No |
| FLA6 | Yes | No | No | No |
| FLA7 | Yes | No | No | No |
| FLA8 | Yes | No | No | No |
| FLA9 | Yes | No | No | No |
| FLA10 | Yes | No | No | No |
| FLA11 | Yes | No | No | No |
| FLA12 | Yes | No | No | No |
| FLA13 | Yes | No | No | No |
| FLA14 | Yes | No | No | No |
| FLA15 | Yes | No | No | No |
| FLA16 | Yes | No | No | No |
| FLA17 | Yes | No | No | No |
| FLA18 | Yes | No | No | No |
| FLA19 | Yes | No | No | No |
| FLA20 | Yes | No | No | No |
| FLA21 | Yes | No | No | No |
| FLA22 | Yes | No | No | No |
| FLA23 | Yes | No | No | No |
| FLA24 | Yes | No | No | No |
| FLA25 | Yes | No | No | No |
| FLA26 | Yes | No | No | No |
| FLA27 | Yes | No | No | No |
| FLA28 | Yes | No | Yes | No |
| FLA29 | Yes | No | No | No |
| FLA30 | Yes | No | Yes | No |
| FLA31 | Yes | No | No | No |
| FLA32 | No | Yes | Yes | No |
| FLA33 | No | No | No | No |
| Native ligand | No | No | No | No |
| Reference ligand | Yes | No | Yes | No |
