# Supplementary material for: Computational insights into flavonoids inhibition of dengue virus envelope protein: ADMET profiling, molecular docking, dynamics, PCA, and end-state free energy calculations
Source: PLoS One. 2025 Jul 9;20(7):e0327862. doi: 10.1371/journal.pone.0327862 (PMC12240381; doi:10.1371/journal.pone.0327862)
Supplement: Attachment — Submitted filename: Response to Reviewers.docx [file pone.0327862.s016.docx]

                                                                                                                                                              01/06/2025

To,

The Academic Editor

**PLOS ONE**

Subject: Responses to the comments and suggestions

Dear Editor,

Herein, we are submitting our revised research manuscript, "**Computational insights into flavonoids inhibition of dengue virus envelope protein: ADMET profiling, molecular docking, dynamics, PCA, and end-state free energy calculations**,**"** for publication in your esteemed journal, **PLOS ONE.** We have addressed the reviewer comments with track changes to the best of our knowledge and effort. We want to express our sincere gratitude for the insightful suggestions and for providing ample time for a thorough revision.

**Journal requirements:**

**1. Please ensure that your manuscript meets PLOS ONE's style requirements, including those for file naming. The PLOS ONE style templates can be found at**

**Response:** We have reviewed the reference list and named the associated files to ensure they are complete and correct according to the journal.

**2. Please note that PLOS ONE has specific guidelines on code sharing for submissions in which author-generated code underpins the findings in the manuscript. In these cases, we expect all author-generated code to be made available without restrictions upon publication of the work. Please review our guidelines at** [**https://journals.plos.org/plosone/s/materials-and-software-sharing#loc-sharing-code**](https://journals.plos.org/plosone/s/materials-and-software-sharing#loc-sharing-code) **and ensure that your code is shared in a way that follows best practice and facilitates reproducibility and reuse.**

**Response:** All relevant data underlying the finding of this study are now publicly available via Figshare repository <https://doi.org/10.6084/m9.figshare.29205689.v1> <https://doi.org/10.6084/m9.figshare.29206427.v1> for the replicate the results reported in this article.

**ACADEMIC EDITOR:**

**Response:** We appreciate the editor's insightful remarks. Your concern has been addressed thoroughly.

**1. The study is appropriately designed and the composure of the manuscript is in order. However, the methodology needs to be presented comprehensively for reproducibility. For instance, the protein preparation steps currently exclude the generation of active binding pocket which is crucial for docking analysis. Secondly, how was the molecular docking analysis validated? What are the pharmacological implications of the molecular interactions observed with reference to the literature? All concerns of the reviewers also require substantial attention of the authors.**

**Response:** We have revised the methods section to comprehensively describe the protein preparation steps. We docked the ligand based on the active site reported on crystal structure of protein. Further, we have detailed our docking validation approach (subsection 3.2).

**2. The study is appropriately designed and the composure of the manuscript is in order. However, the methodology needs to be presented comprehensively for reproducibility. For instance, the protein preparation steps currently exclude the generation of active binding pocket which is crucial for docking analysis. Secondly, how was the molecular docking analysis validated? What are the pharmacological implications of the molecular interactions observed with reference to the literature? All concerns of the reviewers also require substantial attention of the authors.**

**Response:** We have added a Discussion section and more interpretation of the RMSD plots. Comparative analyses with existing literature are now included to support our findings (section 4, subsection 3.5.1)

**Reviewers' comments:**

**Review Comments to the Author**

Please use the space provided to explain your answers to the questions above. You may also include additional comments for the author, including concerns about dual publication, research ethics, or publication ethics. (Please upload your review as an attachment if it exceeds 20,000 characters)

**Response:** Done

**Reviewer #1:**

**Response:** We appreciate Reviewer 1's valuable comments. Your concern has been addressed in the following ways:

**This is a well-motivated and timely study that uses in silico approaches to investigate the potential of flavonoids as inhibitors of the dengue virus envelope (E) protein. The authors have clearly put substantial effort into employing a wide range of computational tools, including molecular docking, molecular dynamics simulations, ADMET profiling, principal component analysis (PCA), and MM/PBSA binding free energy calculations. While the overall methodology is appropriate and the results appear promising, the manuscript requires significant improvements in clarity, language, organization, scientific rigor, and interpretation in order to be suitable for publication.**

**1. Ligand Selection: The criteria for selecting the 33 flavonoids are not clearly explained. Were these compounds chosen based on prior evidence of antiviral activity, structural diversity, or another rationale? Additionally, did the authors evaluate other flavonoids that failed to show positive results? Including this information would strengthen the justification for the selection.**

**Response:** The answer to this question has been included in the introduction part (line no. 71-81)

**2. Docking Validation: The use of an RMSD threshold below 2 Å for docking validation is acceptable. However, a superimposed image of the native and re-docked ligand should be presented in the main manuscript rather than confined to the supplementary information.**

**Response:** The superimposed image of the native and re-docked ligand has been presented in the main manuscript (Fig 2.)

**3. Simulation Parameters: Essential methodological details such as the timestep used, cutoff distances for van der Waals and Coulomb interactions, and the type of thermostat/barostat applied during equilibration must be included to ensure reproducibility.**

**Response:** Key MD parameters, including timestep, cutoff distances, and thermostat/barostat types have been added (subsection 2.4).

**4. Table 2: The list of interacting amino acid residues should be refined by applying a 5 Å cutoff distance to exclude irrelevant or non-specific interactions.**

**Response:** Interacting residues are now filtered using a 5 Å cutoff to improve specificity and relevance (Table 2)

**5. Interpretation of RMSD Values: While the authors emphasize RMSD plots, the manuscript lacks statistical comparisons or a discussion linking these fluctuations to functional or structural implications of ligand binding.**

**Response:** The results section now includes a more robust interpretation of RMSD plots, including comparisons across ligands and implications for complex stability (subsection 3.5.1)

**6. Binding Energy Decomposition: The MM/PBSA energy breakdown is a valuable addition, but the manuscript would benefit from a more detailed biological interpretation. For instance, how do van der Waals and electrostatic interactions contribute to the binding affinity? Does FLA1 replicate or improve upon the interaction pattern of the native ligand βOG?**

**Response:** A more detailed interpretation has been added (subsection 3.6).

**7. Drug-likeness Criteria: The use of the term "drug-likeness" needs further support. It is not enough to rely solely on ADMET predictions: reference to standard frameworks such as Lipinski’s Rule of Five or Veber’s criteria should be included to substantiate the claim.**

**Response:** Thank you for your valuable comments. We have added Lipinski’s rule of five and Verber’s criteria (subsection 3.3).

**8. Figures: The figure organization and presentation need significant improvement. All referenced figures must be embedded within the manuscript itself. For example, Figure 2 ("Two-dimensional projection of the protein-ligand interactions") is cited in the text but the actual image is missing. This undermines the clarity and completeness of the manuscript.**

**Response:** All the figures are presented according to the PLOS ONE guidelines.

**9. Formatting and Writing Style: The authors should carefully review and align their manuscript with the PLOS ONE writing and formatting guidelines, including section organization, figure placement, and citation style. This will ensure the manuscript meets the journal’s structural and stylistic expectations.**

**Response:** The manuscript has been revised thoroughly for consistency with PLOS ONE’s writing and formatting standards.

**10. Broken Link: The manuscript contains an incorrect URL in the Methods section: .<https://pubchem.ncbi.nlm.nhi.gov/> This is a typographical error and should be corrected to:**[**https://pubchem.ncbi.nlm.nih.gov/**](https://pubchem.ncbi.nlm.nih.gov/)**.**

**Response:** The incorrect URL has been corrected to [**https://pubchem.ncbi.nlm.nih.gov/**](https://pubchem.ncbi.nlm.nih.gov/) (line no. 86)

**11. Unmet Research Aim: In the Introduction, the authors claim: “Therefore, effective treatment for dengue is still unknown which is the research gap of this study.” However, the study does not actually propose or validate an effective treatment for dengue. Rather, it identifies a compound (FLA1) with promising in silico interaction potential. The manuscript does not address or close the research gap as stated, and this mismatch between the problem statement and the study’s outcome should be explicitly acknowledged and corrected.**

**Response:** Appropriate changes have been made in the section introduction (line no. 71-81)

**Reviewer #2:** We appreciate Reviewer 2's insightful comments. Your concern has been addressed in the following ways:

**This manuscript explored the potential inhibitory abilities of flavonoids against the dengue virus envelope (E) protein through computational analysis. Despite using a wide range of in silico techniques, such as molecular docking, MD simulations, ADMET screening, and MM/PBSA computations, the manuscript lacks the necessary research justification, methodological depth, and in-depth result analysis and interpretation required for publication at this stage.**

**1. Insufficient Justification of Study: Although the introduction provides a broad overview of the dengue virus and the envelope (E) protein’s role in viral entry, the rationale for selecting flavonoids is poorly developed. The authors mention in general terms that flavonoids possess antioxidant and antiviral properties, but they do not explain why these specific 33 flavonoids were chosen or whether any of them have been previously associated with dengue or other viral infections. This is particularly important given that not all flavonoids have antiviral activity. Moreover, no supporting experimental evidence or literature references are provided to justify the inclusion of these compounds in the screening. Additionally, the overall purpose of the study is not clearly articulated, making it difficult to evaluate whether the study’s design aligns with its intended objectives.**

**Response:** We have clarified the rationale for the selection of flavonoids. Out of *ca.* 3000 flavonoids screened, 33 passed toxicity filters. References to relevant studies supporting their antiviral potential have been added in the Introduction section (line no 73-81)

**2. Absence of a Proper/Standalone Discussion Section: The current Results and Discussion section lacks depth and primarily presents data without insightful analysis or reference to existing literature. The biological significance of the results is not well captured. To strengthen the manuscript, I recommend that the authors include a standalone discussion section that addresses:**

**A. Interpretation of significant findings in the context of previous studies;**

**B. Potential implications for antiviral drug development;**

**C. Limitations of a computational-only approach;**

**D. Future directions such as in vitro/in vivo validation.**

**Response:** We have restructured the manuscript to include a comprehensive Discussion section.

**3. Insufficient Benchmarking: Although the authors refer to n-octyl-β-glucoside (βOG) as the natural ligand targeting the DENV E protein binding pocket, its role is primarily as a site indicator rather than a functionally active antiviral compound. While βOG helps define the binding site in structural studies, it does not substitute for a control compound with validated antiviral potency. To strengthen the comparative framework, the authors should include or substitute known dengue virus E protein inhibitors or experimentally validated antiviral compounds as benchmarks.**

**Response:** We have revised the manuscript to emphasize the use of βOG as a marker of site function instead of an antiviral agent. To enhance the comparative context, we have also added the known reference ligand NITD448 of DENV-E protein inhibitors, as a benchmarking reference compound.

**4. Methodological Gaps and Lack of Adequate Statistical Analysis:**

**The authors assert ligand stability and therapeutic promise based on a single 100 ns molecular dynamics simulation. This is a short timescale, and no replicate simulations were performed to confirm reproducibility. Only conventional MD was conducted, without exploring enhanced sampling methods such as GaMD or metadynamics, which could provide more comprehensive conformational sampling. Additionally, there is no explanation for the choice of CHARMM27 over more updated force fields such as AMBER or CHARMM36m. The MM/PBSA computations, while present, are only superficially interpreted; there is no per-residue decomposition or analysis of specific energetic contributions from residues within the binding site.**

**Response:** Due to our limited computational resources, longer timescale simulations or duplicate runs were not feasible. However, we have enhanced our analysis by including the RDF (subsection 3.5.6). In addition, we have added per-residue decomposition or analysis of specific energetic contributions from residues within the binding site (S8 Fig and S9 Fig). Furthermore, we have justified using the CHARMM27 force field (subsection 2.4).

**5. Neurotoxicity and CNS Relevance Ignored:**

**Although CNS permeability (logPS < -3) is reported, the authors do not explain whether this is advantageous or detrimental. Since dengue virus is known to cause neurological complications, including encephalitis and meningitis, neurotoxicity is a relevant concern. It remains unclear whether BBB impermeability restricts the therapeutic potential of the screened compounds or if it is beneficial—for example, to prevent off-target CNS effects. The authors should explicitly address the CNS relevance of their findings, particularly in the context of drug safety and treatment efficacy.**

**Response:** We have now added the implications of CNS permeability in the context of dengue pathology and drug safety (subsection 3.3).

**6. ADMET Interpretation Is Superficial: Although ADMET profiling is included, the manuscript offers minimal interpretation of the data. For example, toxicity class designations and plasma protein binding (PPB) values are reported without explaining their implications. What does a “good PPB” mean in the context of therapeutic safety or drug efficacy? Are the observed toxicity classes acceptable for antiviral use? These factors are essential for assessing the safety and drug-likeness of the candidates. Furthermore, there is no comparison between the flavonoid candidates and established antiviral medications. Conclusions like "CNS-impermeable" or "non-sensitizing" are given without elaborating on the significance of these characteristics for dengue treatment. ADMET results must be interpreted in a pharmacological context, not merely listed.**

**Response:** We have expanded the interpretation of ADMET properties, including plasma protein binding and CNS permeability, with reference ligand (NITD448) (subsection 3.3).

Summary of major changes

1. Revised methodology;
2. Added benchmarking;
3. Clarified ligand selection and study aims.
4. Introduced a standalone Discussion section;
5. Added RDF section;
6. Improved statistical and pharmacological interpretation of MD and ADMET, binding free energy;
7. results;
8. Enhanced figure quality and formatting;
9. Corrected all typographical and citation issues.

We sincerely appreciate the feedback that has helped to refine the manuscript and enhance its quality concerning the scientific content, along with the mode of presentation.

Thank you once again for your valuable input.

Sincerely,

Authors
